# Traffic coordination by reducing jamming attackers in VANET using probabilistic Manhattan Grid Topology for automobile applications

**DOI:** 10.1038/s41598-024-58240-2

**Published:** 2024-04-10

**Authors:** G. B. Santhi, Suma Sira Jacob, D. Sheela, P. Kumaran

**Affiliations:** 1 Department of Computer Science and Engineering, New Prince Shri Bhavani College of Engineering and Technology, Chennai, India; 2 Department of Artificial Intelligence and Data Science, Sri Krishna College of Technology, Coimbatore, India; 3 Department of Electronics and Communication Engineering, Saveetha School of Engineering, Saveetha Institute of Medical and Technical Sciences, Chennai, India; 4 Department of Mechanical Engineering, College of Engineering, Wolaita Sodo University, Wolaita Sodo, Ethiopia

**Keywords:** Vehicular adhoc network, Fuzzy C-means algorithm, Modified fisheye state routing algorithm, Modified Manhattan grid topology, Particle swarm optimization, Engineering, Mathematics and computing

## Abstract

In recent years Intelligent Transportation System (ITS) has been growing interest in the development of vehicular communication technology. The traffic in India shows considerable fluctuations owing to the static and dynamic characteristics of road vehicles in VANET (Vehicular Adhoc Network). These vehicles take up a convenient side lane position on the road, disregarding lane discipline. They utilize the opposing lane to overtake slower-moving vehicles, even when there are oncoming vehicles approaching. The primary objective of this study is to minimize injuries resulting from vehicle interactions in mixed traffic conditions on undivided roads. This is achieved through the implementation of the Modified Manhattan grid topology, which primarily serves to guide drivers in the correct path when navigating undivided roads. Furthermore, the Fuzzy C-Means algorithm (FCM) is applied to detect potential jamming attackers, while the Modified Fisheye State Routing (MFSR) Algorithm is employed to minimize the amount of information exchanged among vehicles. Subsequently, the Particle Swarm Optimization (PSO) algorithm is developed to enhance the accuracy of determining the coordinates of jamming attackers within individual clusters. The effectiveness of the outcomes is affirmed through the utilization of the Fuzzy C-Means algorithm, showcasing a notable 30% reduction in the number of attackers, along with the attainment of a 70% accuracy rate in this research endeavor.

## Introduction

A vehicular Ad-hoc Network (VANET) is a network in which vehicle nodes can communicate in a multi-hop fashion with each other on the road. It provides communication between vehicles and roadside units to provide safe transportation^1^. VANET has several features such as bigger node size, influence on energy, and computational ability as compared with wireless sensor networks (WSN). The attackers create different types of attacks for all network users, which is the biggest issue in VANET. The safe and sound applications are most beneficial in VANET. They are time-critical and need data transmissions from one vehicle to another vehicle at the exact time. In timing attacks, when malicious vehicles receive a message, they do not forward it as normal but add some timeslots to the original message leading to delay.

The main key issue in the implementation of VANET is providing secure vehicular communication^2^. In VANETs, some critical event information must be distributed quickly and reliable manner. It is a challenge to communicate critical messages timely and reliably to targeted vehicles in VANET due to the dynamic nature of the network.

## Literature survey

The various aspects of Vehicular Ad-Hoc Networks (VANETs) have been covered in survey papers, and it's essential to have comprehensive reviews of the literature to understand the current state of research in this field.The stability of the cluster member in a cluster is reflected by the duration of the particular cluster which allows for the evaluation of the reliability of the inter-cluster links. The maintenance time, head, and members are stable in a stable cluster^2^. In this work, switching frequency was effectively estimated by the duration of the cluster.

In multi-hop cluster architecture obtaining accurate motion information is difficult^3,4^. Hence it is rigid to determine the cluster head nodes in the multi-hop range and the maximum number of broadcasting packets caused by increasing the network overhead. The authors reported the security analysis in their Dual-protected ring signature algorithm. It shows the different categories of security requirements for VANETs. The vehicle broadcasts every message and these are frontier to the protected identifier, timestamp, and identity-based signature. By applying the Fuzzy C-means algorithm the messages within the RSU are clustered using various strategies, leading to a reduction in the volume of packet transmissions within the VANET region^5^. In this work, researchers improve collision avoidance measures in the aftermath of an incident using a Fuzzy C-means clustering algorithm.

An energy-efficient and QoS-aware routing technique allows for control of excessive dissemination of data traffic across the networks. In this work, authors improved overall real-time performance and control of the data dissemination rate across vehicular networks. Although exchanging up-to-date information has provided a successful model of intelligent automotive systems, it may raise a problem in network bandwidth, response time, and power consumption^6^. The integration of V2I transmissions and V2V re-transmissions operates as follows: initially, Roadside Units (RSUs) disseminate alert messages, and subsequently, chosen relay vehicles retransmit these messages following a customized delay^7^. Furthermore, the SDN controller defines broadcast zones, within which relay vehicles can retransmit messages to ensure coverage for all vehicles in the specified region, especially those in areas with limited or weak signal coverage.

A threshold-based separation technique is used to differentiate the normal and abnormal behavior of traffic flows^8^. Each user makes progress rapidly, which leads the way to the corresponding distance between two dynamic users. The users of a VANET, access the network randomly, which leads the way to the short-lived link. Data dissemination strategies in VANET help improve safety, efficiency, and comfort by reducing the delivery delay time and ensuring the reliability of message delivery. The motivation behind this work^9^ is to provide he audience with a comparative analysis of delay-tolerant and delay-sensitive data dissemination in VANET.

With the increasing number of vehicles and the density of information, there is a growing need for efficient data exchange to support various applications. In this article^10^, the Vehicle-Consensus Routing Management Scheme (VCRMS) is designed to ensure equitable roadside assistance for drivers. This scheme leverages nearby vehicle data to make informed choices regarding infrastructure selection and traffic management. Deep learning techniques are employed to analyze both infrastructure and vehicle information, extracting patterns to make more consistent decisions. As the number of vehicles within the network grows, traffic congestion becomes a prevalent issue. In response to this challenge, a novel traffic management system, known as CoNeCT (Collaborative Information Sharing for Vehicular Ad hoc Networks with Predictive Congestion Control), has been deployed^11^. The primary objective of CoNeCT is to facilitate collaborative efforts among vehicles to analyze, predict, and effectively manage congestion. This system has been specifically engineered to reduce the volume of messages by introducing an innovative approach to assessing road segment loads, ultimately enhancing the classification of traffic flow and improving traffic circulation.

The implementation of a congestion control algorithm based on transmission power, utilizing a Markov decision process (MDP) and its resolution through a Q-Learning algorithm.This work^12^ is to to enhance the efficiency of VANETs by proficiently handling channel load and mitigating congestion through the maintenance of a channel busy ratio (CBR) close to the critical threshold of 0.6The algorithm incorporates the adjustment of transmission power levels to reduce the channel busy ratio while still ensuring a high level of awareness concerning nearby vehicles. Traffic parameters, notably vehicular density, demonstrate a noticeable statistical variation when compared to situations without accidents. Building upon this observation, an algorithm for detecting false messages is introduced. It leverages the vehicles in motion as witnesses to gather traffic-related data, using their observational data as evidence to support a traffic flow model. The methodology relies on Bayesian theorem principles to compute the likelihood of various traffic scenarios, ultimately estimating the real traffic conditions to ascertain the validity of the reported accidents^13^.

IoV technologies and tools play a crucial role in establishing the Internet of Vehicles and addressing traffic regulations, utilizing SUMO (Simulation of Urban Mobility) for road traffic design and simulation. The authors of this study^14^ aimed to make a valuable contribution by refining the traffic control system model. In this research, two vehicular congestion control models were scrutinized to determine the most efficient and optimized option. The findings revealed that an IoV-based model operating within vehicular clouds outperforms the traditional model, offering compelling reasons for enhancing the overall network system.

## Proposed methodology

The main goal of this research is to minimize injuries resulting from vehicle interactions in mixed traffic conditions on undivided roads. This objective is accomplished by implementing the Modified Manhattan grid topology, which primarily serves to provide guidance to drivers on the correct path while navigating undivided roads. Additionally, the Fuzzy C-Means algorithm is used to identify the number of potential jamming attackers, and the modified fisheye algorithm is utilized to reduce the volume of information exchanged between them. Following that, the Particle Swarm Optimization (PSO) algorithm^15^ is formulated to calculate more accurate coordinates for jamming attackers within each cluster.

### Modified VANET architecture

**Figure 1:**
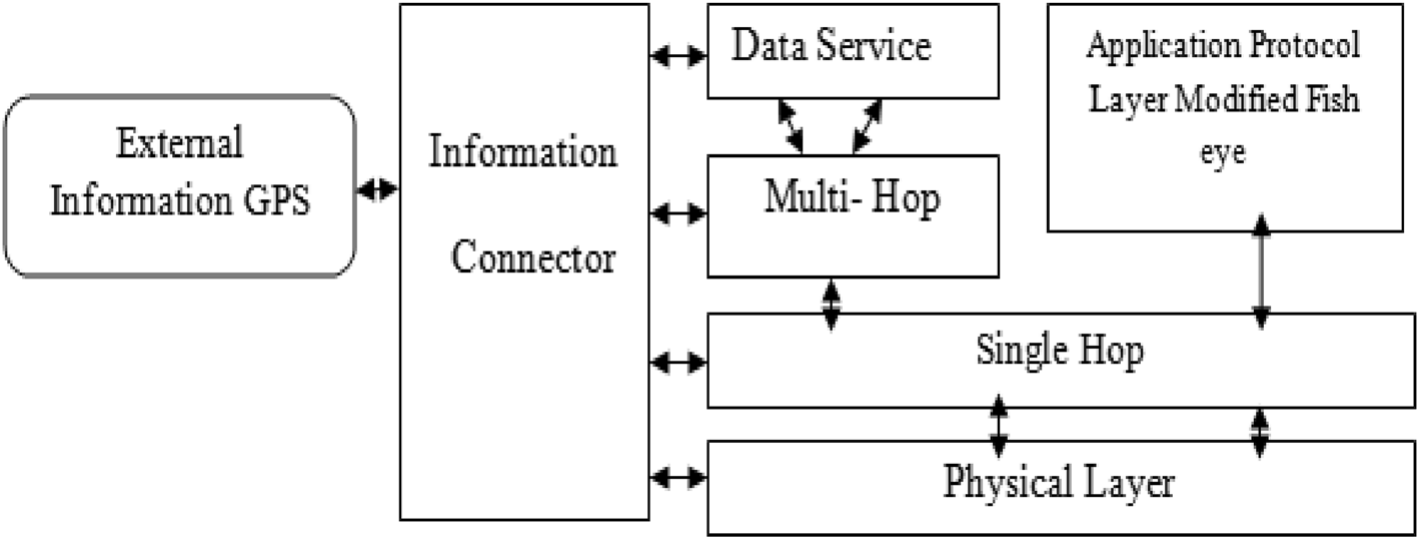
Modified VANET architecture.

Figure 1 shows the modified VANET architecture which integrates blocks such as data service, information connector, single and multihop, and application protocol using modified fish eye blocks.

The external information connector utilizes Global Positioning Services (GPS) to collect location-specific data, which is subsequently stored within the connector. This data encompasses information about roads and the routing map. The transmission of data and control packets occurs through the physical layer. In a single-hop configuration, each road is linked individually, whereas in a multi-hop setup, individual roads are clustered together to establish the network. The Fish Eye routing algorithm is applied to handle short-range traffic timing in Ad hoc networks, while the Modified Fisheye State Routing (MFSR) algorithm is employed for longer-distance coverage. The modification in the protocol is explained below^7^. A zone that is enclosed by hop distances. Since Fish Eye State Routing is a linked state-based routing protocol directional link states can be included in the FSR update messages. The fisheye approach translates to maintaining the accurate distance in routing and path quality information about the immediate neighborhood of a node^16^.

In this research work, the traffic region is divided into two types namely, jammed region, and unaffected jammed region. The traffic controller helps in detecting and controlling the traffic flow in the jammed region. The Fuzzy C-Means algorithm is employed to address and mitigate the presence of jamming attackers, effectively determining their quantity or count within the VANET. Jamming attackers are individuals or entities that deliberately disrupt communication within the network by emitting interfering signals or noise. These attackers use jamming techniques to overpower or interfere with legitimate communication between vehicles or between vehicles and infrastructure components, such as roadside units or access points. Jamming attackers aim to create chaos and disrupt the normal functioning of the VANET, potentially causing accidents or hindering the exchange of critical safety-related information among vehicles. Subsequently, the Particle Swarm Optimization (PSO) algorithm is devised to compute more precise coordinates for jamming attackers within each cluster.

Clustering plays a pivotal role in the realm of Vehicular Ad Hoc Networks (VANETs), where multiple vehicles unite to establish groups predicated on shared characteristics. Within a clustered vehicular environment, the vast network of vehicles is regarded as an amalgamation of smaller networks or clusters. The formation of these clusters hinges on various metrics, including but not limited to average relative velocity, acceleration, position, direction, vehicle degree, vehicle density, transmission range, and more. The selection of a Cluster Head (CH) is based on the assessment of stability among participating vehicles, with the remaining vehicles assuming the role of Cluster Members (CMs). In this research study, clustering is employed in conjunction with the Fuzzy C-Means algorithm to forecast vehicle movements by leveraging fuzzification techniques. Additionally, the aim is to diminish the count of potential attackers in the system^17,18^.

### Fuzzy C-mean algorithm

The fuzzy C-Mean algorithm is a hard clustering algorithm that makes sure that the vehicular node either belongs to a cluster or not. However, fuzzy clustering allows each vehicular node x_k_ to belong to several clusters with associated membership degree values from 0 to 1. The fuzzy C-Means algorithm generates fuzzy partitions and prototypes for any set of numerical data. In this research work, a circular region is centered at the jammer’s location. The jammed region contains several jammed points, and Each jammed point locates its position closer to its jamming source. Hence, the Fuzzy C-Means algorithm is used to perform a sequence of clustering processes to X = {x_k_} at C = 1,2,3,………, where C is the number of clusters.Ci = {x_1_^i^ ,x_2_^i^,……..,x_n_^i^} to represent the ith cluster, i = 1,2,3,….c, where n =|C_i_| FCM algorithm is based on the deprecation of an objective function is shown in Eq. (1) called C-Means Function

1 $${J}_{FCM }\left(X,U,V\right)= \sum_{i=1}^{C}\sum_{k=1}^{n}{u}_{ik }^{m} {d}^{2} \left({x}_{k , }{v}_{i}\right)$$

where *X* is the matrix of the input data points, *V* = [*v*_*1,*_*v*_*2*_* . . . v*_*c*_*]* is the matrix of the cluster centers to be determined, U is the matrix of the fuzzy membership degrees, n is the number of input data points, u_ik_ ϵ [0,1] is membership degree value of the data point x_k_ in the kth cluster v_i_, and v_i_ is the centroid of the cluster I, m > 1 is the fuzzy index which controls the fuzziness of the resulting partition and d is the distance between point x_k_ to v_i_.

### Centroidal coordination beam routing system

**Figure 2:**
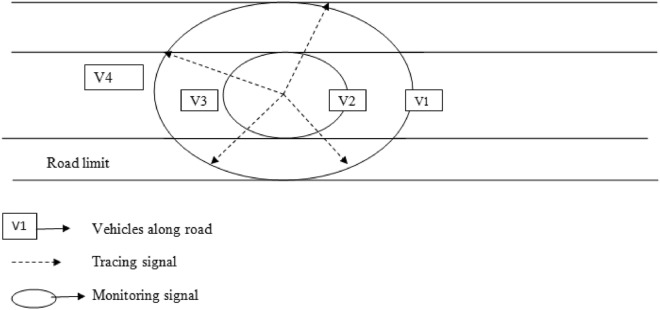
Centroidal coordination beam routing system.

The topology for the centroidal coordination beam routing system is shown in Fig. 2.

Tracking limits are established based on the central point of the coordinate system, with two circles encompassing the outermost boundaries of the circular region^19^. Within this area, vehicles v1, v2, v3, and v4 navigate and move. In Fig. 2, node v2 initiates message transmission to the nearest node, v3, and subsequently to v4, which is within its neighbor list. The distances, dv4 (from v3 to v4) and dv3 (from v3 to v2), are computed as described in Eqs. (2) and (3). The permissible communication distance, denoted as dmax, is determined using Eq. (4). The area of overlap between two circles is defined: one centered at v4 with a radius of dmax and the other centered at v2 with a radius equal to the maximum communication distance. This overlapping region is termed the allowable communication area, denoted as "S." Each node within S satisfies two conditions: it is not only in proximity to the destination node, v4 but also falls within the communication range of node v2. Consequently, these nodes are deemed suitable candidates for selection as the next-hop nodes for v2.

2 $${d}_{ {v}_{3} {v}_{4}}=\sqrt{{(X}_{{v}_{4}}-{X}_{{v}_{3}}{)}^{2}+ ({Y}_{ {v}_{4}}-{Y}_{{v}_{3}}{)}^{2}}$$

3 $${d}_{ {v}_{2}{v}_{3}}=\sqrt{{(X}_{{v}_{2}}-{X}_{{v}_{3}}{)}^{2}+ ({Y}_{{v}_{2}}-{Y}_{{v}_{3}}{)}^{2}}$$

4 $${d}_{max}= {d}_{ {v}_{3} {v}_{4}}+ {\lambda Xd}_{ {v}_{2}{v}_{3}}$$

Evidently, the parameter λ influences the size of S. With a high λ value, S expands in size, resulting in the selection of nodes in close proximity to v2 as the next hop within S. However, this choice may lead to an increase in the number of hops required for these nodes to reach v4. Conversely, when λ is set very low, S contracts in size, leading to the preference for nodes closer to v4 as the next hop within S. In this scenario, the distance from v2 to these selected nodes may increase, potentially degrading link stability and causing an uptick in packet loss.

### Signal flows in traffic coordination system

**Figure 3:**
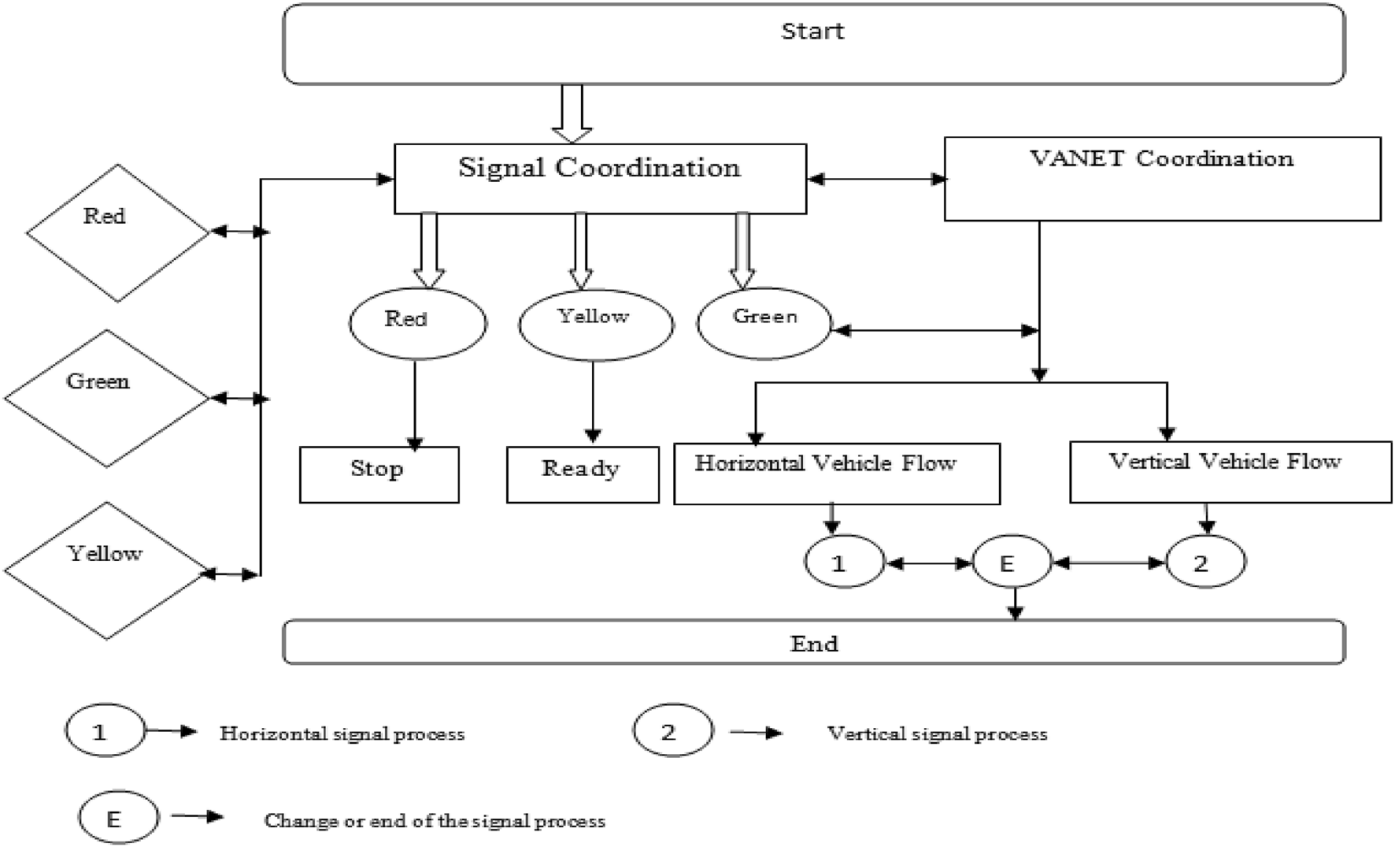
Signal flow for proposed work.

The traffic regulation system, referred to as the Signal Flow Coordination System, plays a crucial role in managing traffic. This system has been implemented at road junctions to oversee and control the movement of vehicles, aiming to reduce accidents and minimize delays at these intersections. Figure 3 illustrates the traffic coordination signal flow as part of the proposed approach.

The Signal Flow Coordination System, often referred to as a Traffic Signaling System, is a device placed at intersections where horizontal and vertical roads meet. Its function involves signaling safe times for driving or walking by employing a universally understood color scheme comprising red, yellow, and green hues. Within the signal coordination system, the color 'Red' indicates the requirement to halt completely before reaching the stop line, emphasizing the importance of avoiding intersection congestion. The 'Yellow' signal prompts readiness for movement, while the 'Green' light permits vehicles to move both horizontally and vertically. This synchronization between horizontal and vertical vehicle flow harmoniously integrates with the coordination of the Vehicular Ad-Hoc Network (VANET). Upon the green signal being displayed in the signal coordination system, the VANET coordination system enables vehicle movement either horizontally or vertically along the road, continuing until the program's completion.

### Modified Manhattan grid topology

**Figure 4:**
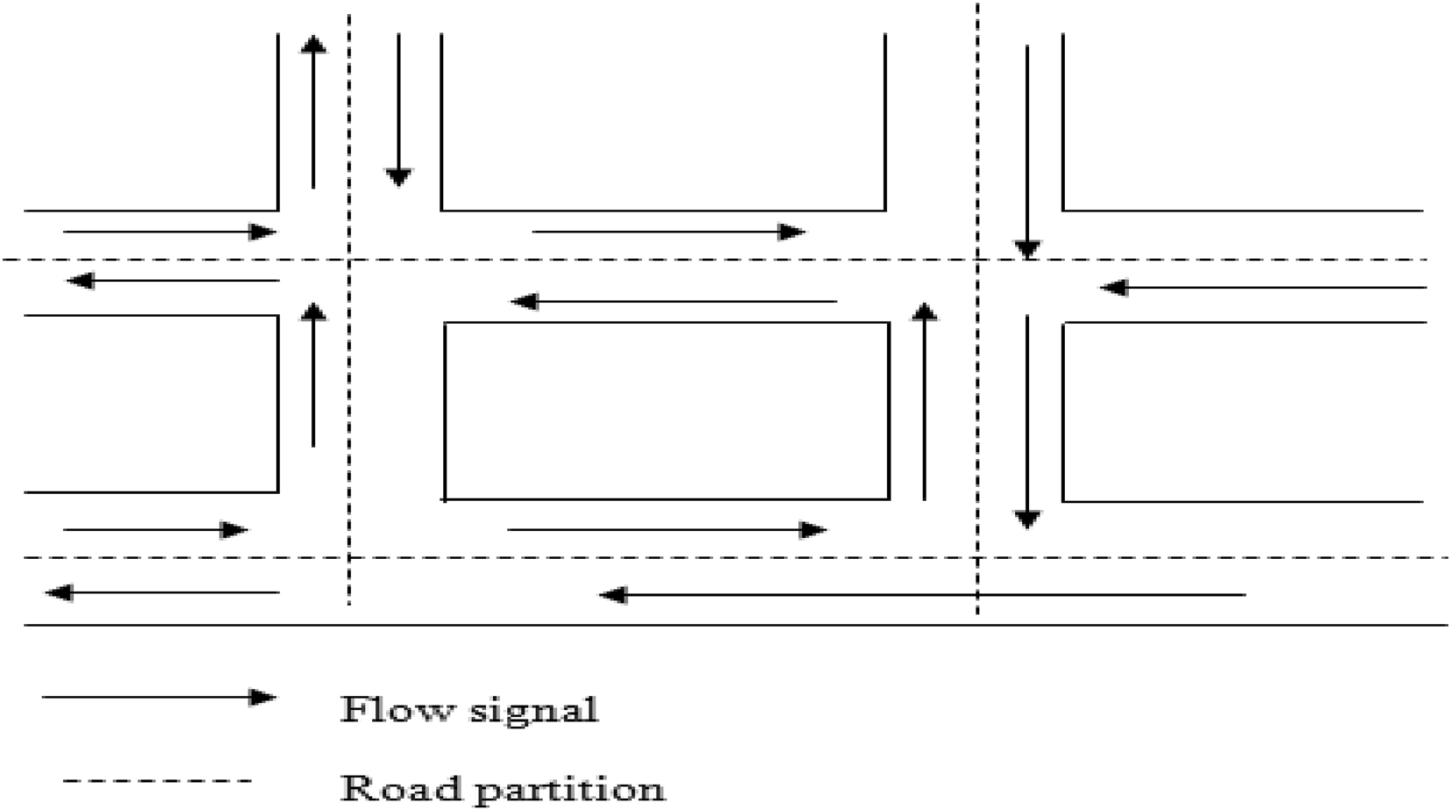
Modified road topology with flow signal.

In this research project, several sensible alterations were implemented in the Manhattan Road topology. The primary modification involves the introduction of an additional parameter, specifically the minimum speed requirement for a vehicle node. This addition proves beneficial because vehicle speeds can approach values very close to zero. The model defines that speed should be updated at certain distance intervals, and without this parameter, there could be extended periods of extremely slow node movement. In this research project, we focus on city intersections, where each intersection relies on three distinct traffic flow directions: left, right, and straight movements. The modified road topology with the flow signal and road partition is shown in Fig. 4.

At this intersection, two parallel roads intersect with two vertical roads, and traffic flows are indicated by arrows. The coordination beams effectively encompass all possible routes, enabling traffic monitoring. In this probabilistic Manhattan grid topology, vehicles traverse both horizontal and vertical streets in their designated lanes. At intersections between horizontal and vertical roads, vehicles follow a specific probability distribution^20^. While this probabilistic method may not be suitable for highway systems, it offers a flexible means of altering the direction of vehicular nodes and upholds geographical constraints on vehicular node mobility.

**Figure 5:**
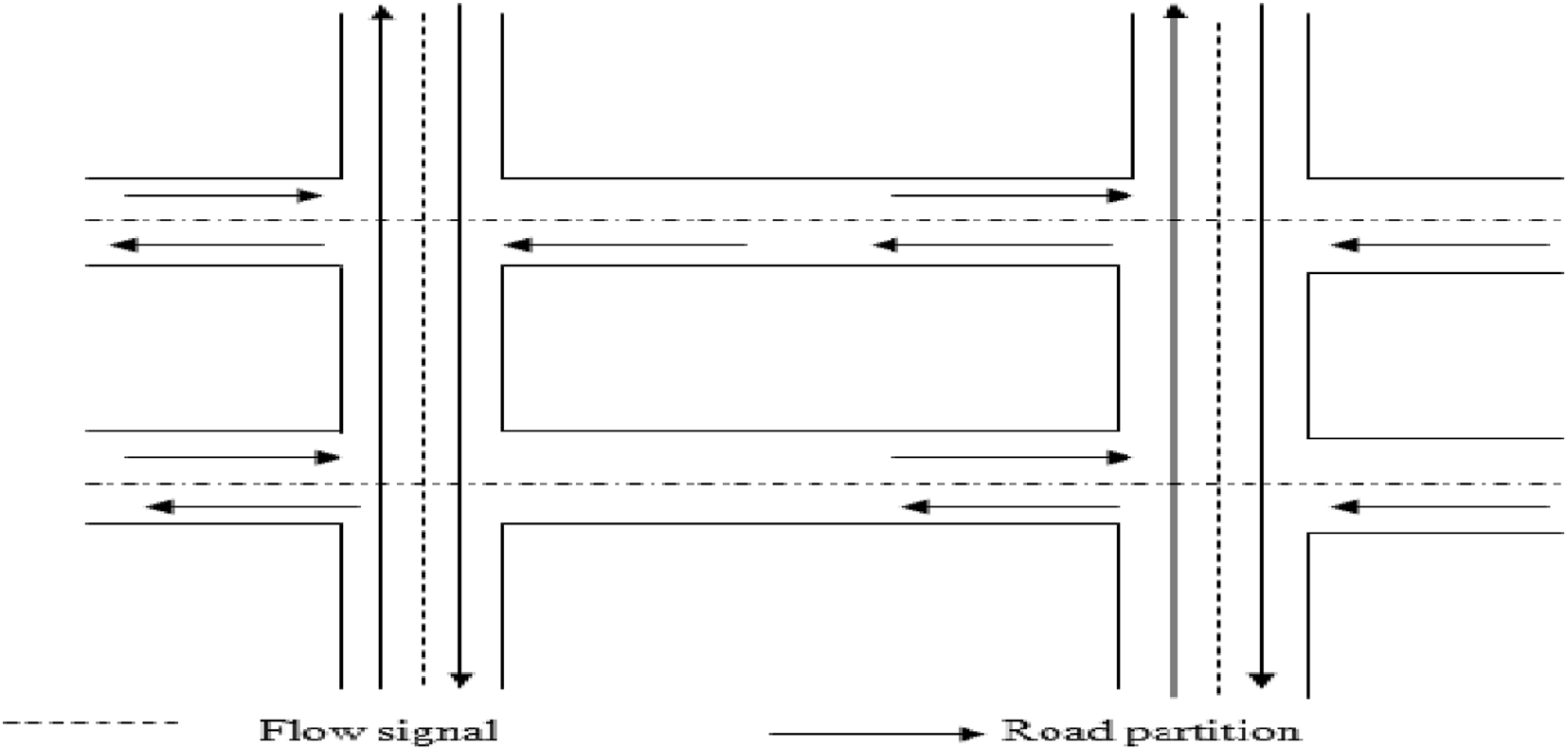
The probabilistic approach of manhattan grid topology model.

The Probabilistic approach of the Manhattan grid topology model is shown in Fig. 5. In this model, a probabilistic strategy is employed to determine vehicle movements, where each vehicle consistently travels in a single direction at each intersection. Specifically, there is a 50% probability of proceeding straight ahead and a 25% probability each of turning left or right.

**Figure 6:**
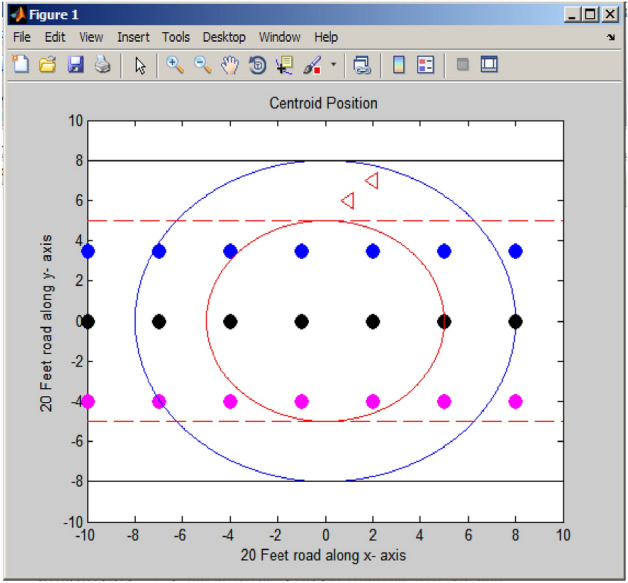
Centroidal coordination beam.

In Fig. 6, there are two coordination beams: one represented by a red circle (Beaconing circle) and the other by a blue circle. These circles denote the coverage areas positioned at the center of the road. The red line and the black line, on the other hand, mark the endpoints of the vehicle's Journey.

The dots such as blue, black, and pink represents the vehicles traveling along the side of the road. The 20 feet road represents the x and y direction of the graph. The two red color triangles pass the attackers' related information to the roadside unit. The probability of a vehicle traveling along the roadside can be determined using the weight-based centroids in the Fuzzy C-Means (FCM) algorithm^21^. It is shown in Fig. 6.

The Manhattan path-pair Determination algorithm is used to determine the next node whose Positive-Path Counter and Negative-Path Counter are both greater than 0 as the node in the vertical movement (X_d_, Y_d_) and in the horizontal movement (X_d_, Y_d_), respectively. Let us assume that the source node (X_s_, Y_s_)is (1,1) and the destination node is node (M, N), The Manhattan grid topology algorithm is shown below.

The Manhattan path-pair Determination algorithm.
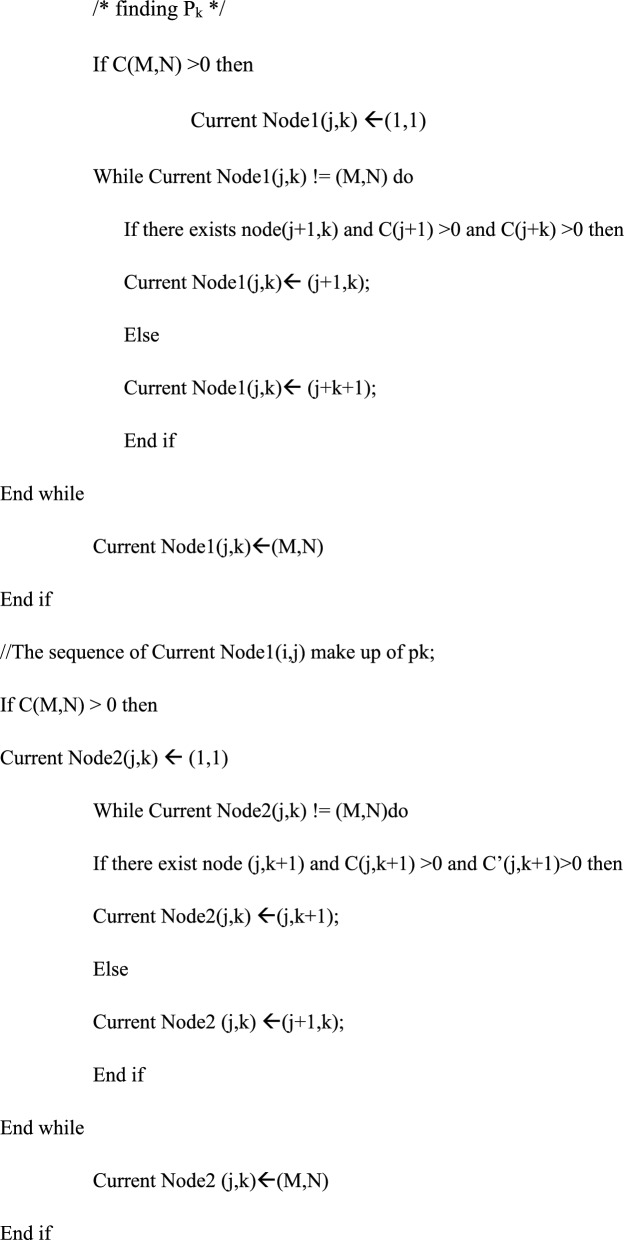


The sequence of Current Node2 (j,k) makes up P_k_;

The best way to determine the accessible next node in the X+ or Y+ direction in the Manhattan Grid topology Algorithm is to let P_k_ and P_k+1_ separate from each other as far as possible. When (X_d_, Y_d_) is in other directions of (X_s_, Y_s_), the similar path pair can easily be deduced.

**Figure 7:**
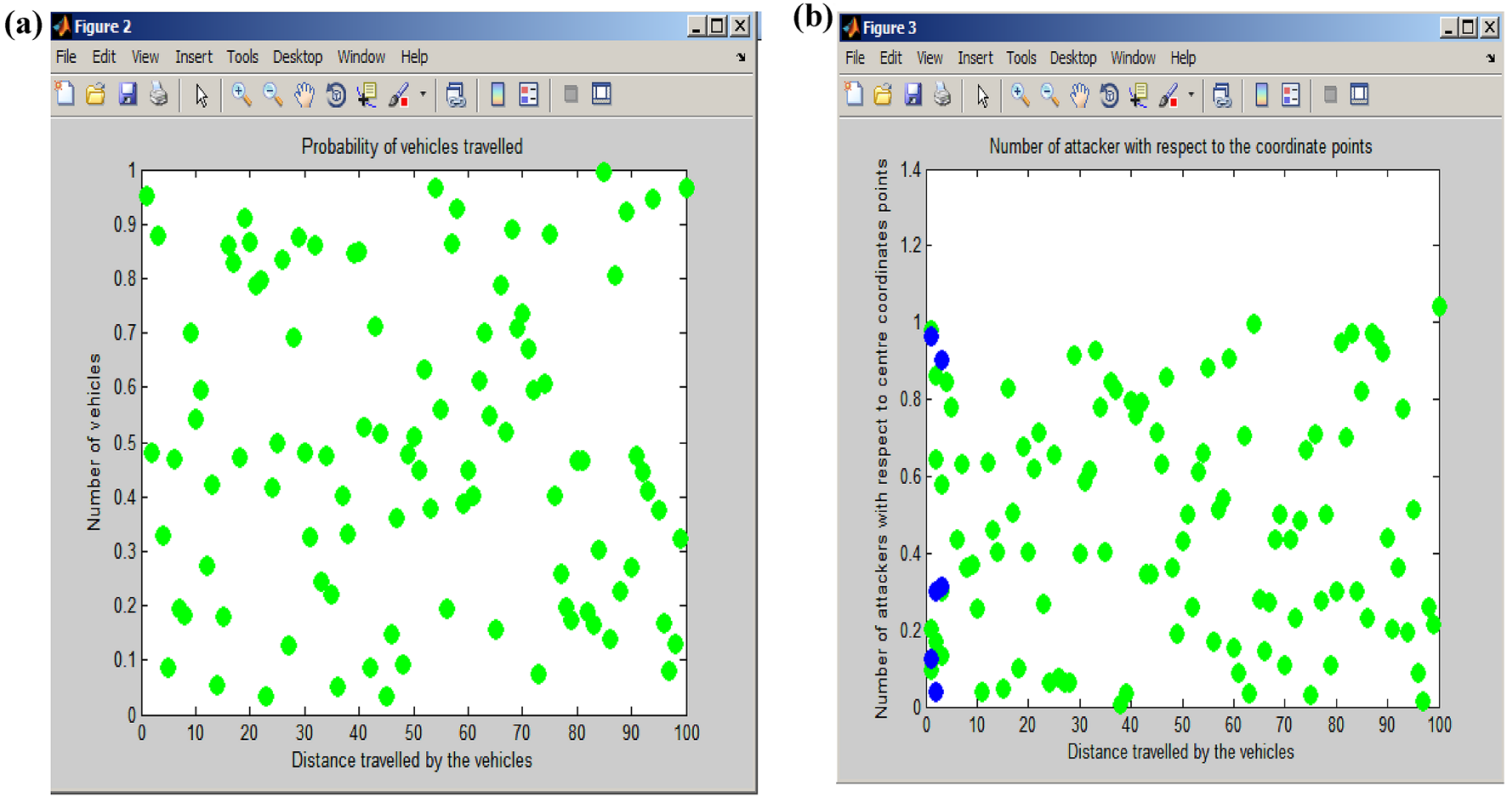
Probability of vehicle and attackers. (**a**) Probability of vehicle travelled. (**b**) Probability of attacker.

The Beaconing circle and Coverage rate serve as indicators for assessing the likelihood of vehicles traveling along the roadside. Vehicles can travel a distance of up to 100 m along the x-axis of the road, with a probability of travel ranging from a minimum of 0.1 to a maximum of 1. During the busiest peak hour, a maximum of one vehicle is expected to travel along the road, while during other times, the number of vehicles on the road may vary, typically falling between 0.5 and 0.9, indicating lower traffic compared to the peak hours. In this particular implementation, a dataset comprising 100 vehicles has been employed. Figure 7 illustrates the probability of vehicles and attackers. The probability of vehicles traveling is shown in Fig. 7 (a). In this context, the x-axis represents the distance covered by vehicles, while the y-axis represents the count of vehicles traveling on the road. Figure 7b illustrates the predicted attackers using the PSO algorithm^22^, with blue dots indicating the presence of attackers and green dots representing the number of vehicles traveling alongside coordination points. Specifically, the x-axis denotes the distance covered by vehicles, and the y-axis corresponds to the presence of attackers in relation to the coordination points along the road.

Table 1 shows the attackers on each iteration.

**Table 1:** Attackers on each iteration.

S.No.	Iteration count	Attackers tracking
1	Iteration count = 1,	obj. fcn = 2.351306
2	Iteration count = 2,	obj. fcn = 1.680048
3	Iteration count = 3,	obj. fcn = 1.524155
4	Iteration count = 4,	obj. fcn = 1.493228
5	Iteration count = 5,	obj. fcn = 1.489828
6	Iteration count = 6,	obj. fcn = 1.488405
7	Iteration count = 7,	obj. fcn = 1.487489
8	Iteration count = 8,	obj. fcn = 1.486888
9	Iteration count = 9,	obj. fcn = 1.486496
10	Iteration count = 10,	obj. fcn = 1.486242
11	Iteration count = 11,	obj. fcn = 1.486079
12	Iteration count = 12,	obj. fcn = 1.485974
13	Iteration count = 13,	obj. fcn 1.485906
14	Iteration count = 14,	obj. fcn = 1.485863
15	Iteration count = 15,	obj. fcn = 1.485836
16	Iteration count = 16,	obj. fcn = 1.485819
17	Iteration count = 17,	obj. fcn = 1.485808
18	Iteration count = 18,	obj. fcn = 1.485800

### Beam form coordination in proposed work

**Figure 8:**
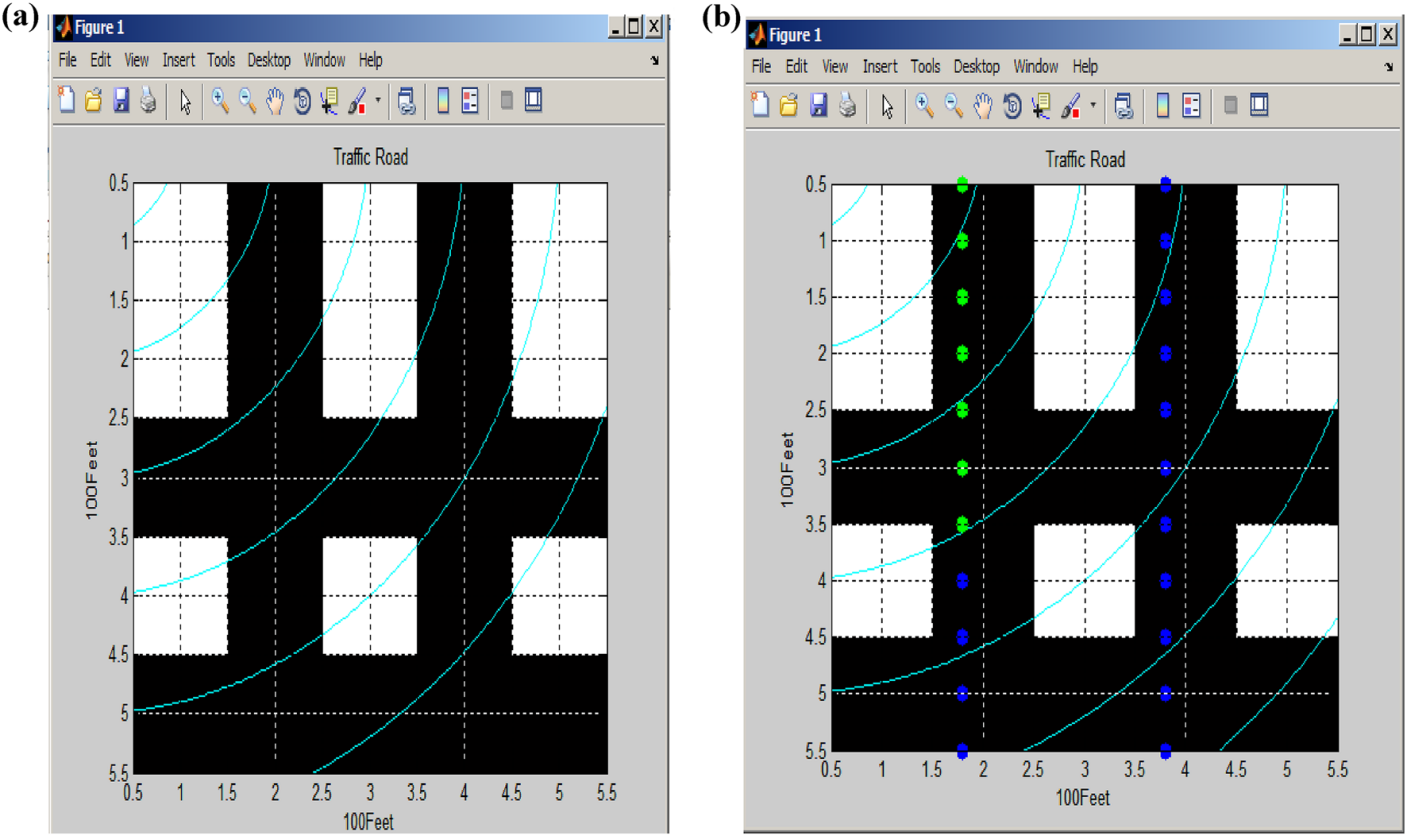
(**a**) Modified VANET with beam forming coordination. (**b**) Vertical flow roads.

Figure 8a shows the modified beam forming coordination activity with road topology. The x-axis corresponds to a 100-foot road, which includes two parallel roads and two vertical parallel roads. The coordination beam effectively covers the entirety of this road. In Fig. 8b, the vertical signal flow, facilitated by the coordination beam, is depicted. Green dots symbolize vehicles moving in one direction along the road, while blue dots represent the direction of vehicle flow in the opposite direction.

### Modified fisheye state routing (MFSR)

**Figure 9:**
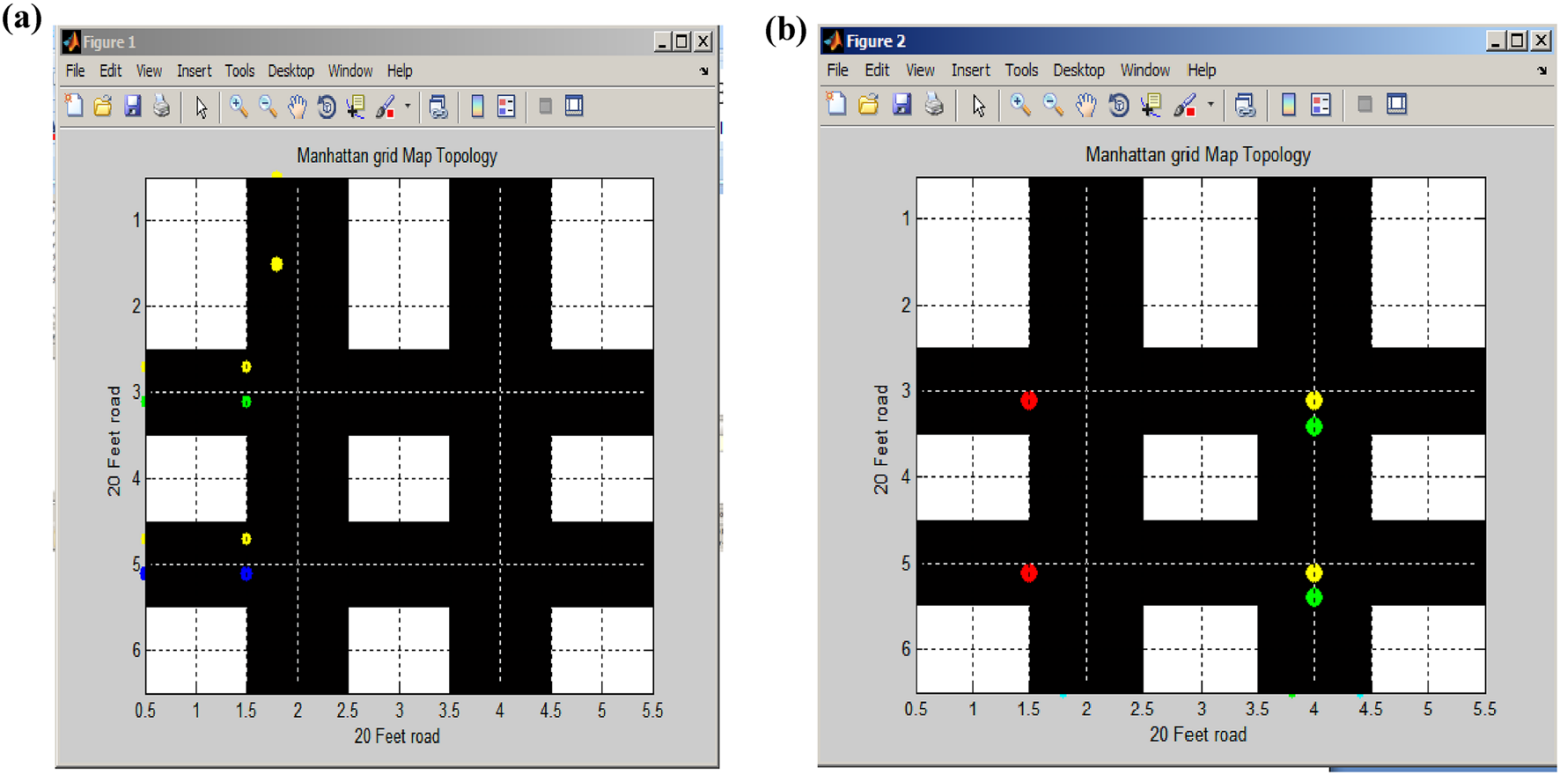
Vertical and horizontal flow routing. (**a**) Proposed Manhattan grid map topology. (**b**) Traffic signal routing.

The coordination beam oversees traffic flow in both horizontal and vertical directions of vehicular movement. This beam is established through the utilization of the Modified Fisheye State Routing (MFSR) algorithm. The fisheye routing algorithm is designed to reduce the amount of information needed to represent graphical data effectively^23^. It operates on a principle similar to the way a fish's eye captures high-detail pixels near its focal point, with detail gradually diminishing as the distance from the focal point increases^24^. In this case, the focal point spans from one corner of the road to the opposite corner. Figure 9 provides a visual representation of routing for both vertical and horizontal flows in all directions.

The road's dimensions are represented along both the x-axis and y-axis, covering a 100-foot span. In Fig. 9a, we observe the newly devised Manhattan grid topology road map, specifically designed to minimize vehicle flow. This map combines two parallel regular roads, one horizontal and one vertical, to facilitate the routing process. In Fig. 9b, the Manhattan grid map topology is depicted, where signal routing is conducted through conventional traffic signals. In this system, the red signal signifies a stop, green indicates the clearance to proceed, and yellow serves as a preparation signal .

**Figure 10:**
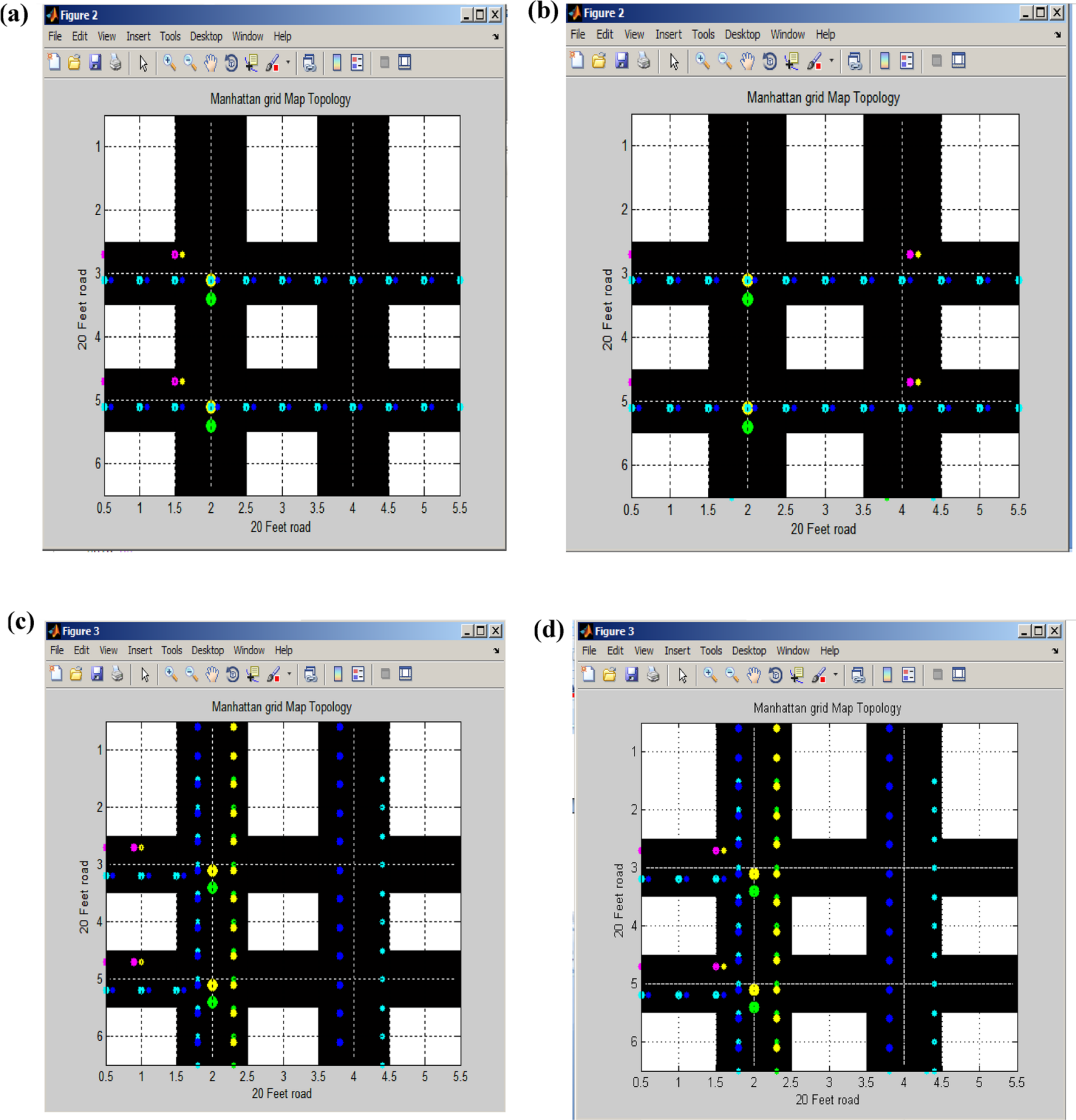
(**a**) First half horizontal routing signal. (**b**) Second half horizontal routing signal. (**c**) First half vertical routing signal. (**d**) Second half vertical routing signal.

Figure 10a,b illustrates the initial and latter segments of the horizontal routing signal. The cyan and blue segments represent unobstructed signal flow in the two horizontal road sections, while the pink and yellow segments depict signal flow in the first half of the horizontal section^25^.

Figure 10c displays the vertical signal flow in both the vertical direction and the first half of the horizontal traffic signal. Similarly, Fig. 10d presents the vertical signal flow in both the vertical direction and the other half of the horizontal traffic signal.

**Figure 11:**
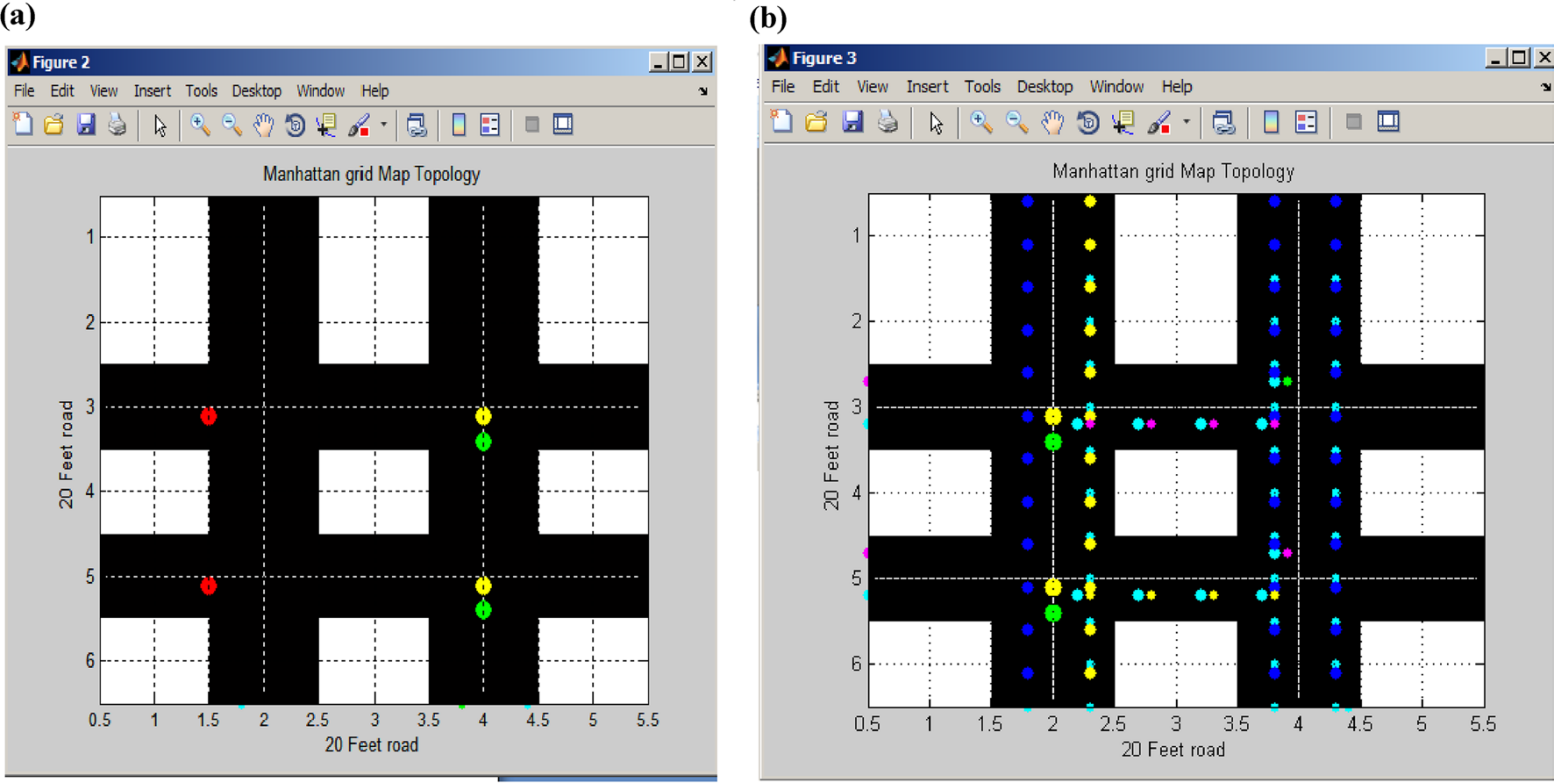
(**a**) Traffic routing of the vertical signal. (**b**)Traffic routing of the first half vertical signal.

Figure 11a illustrates the second routing of vertical signal flow originating from a standard horizontal signal, while Fig. 11b depicts this signal transformation within the first and second halves of the signal. The horizontal road is divided into 0.5, 1, 1.5, 2, and 2.5 feet segments, representing the initial half of the signal timing. Subsequently, the remaining half of the horizontal signal extends from 3, 3.5, 4, 4.5, and 5.0 to 5.5 feet along the 20-foot road. In the vertical road section, signal flow covers the range from 1.5 to 2.5 feet, and another portion of the vertical signal spans from 3.5 to 4.5 feet. The central point of signal convergence occurs at the juncture of the two parallel vertical roads^26^. Table 2 provides the calculations for determining the number of potential attackers.

**Table 2:** Calculation for number of attackers.

Distance	Speed (km/h)	Number of attackers
300	30–50	50
1000	70–100	426
5000	> 100	450

## Simulation scenrio exploration

In this section, there is an explanation for the investigational setup and the outcomes of our research done in the Vehicular ad-hoc network and the proposed Modified Fisheye State Routing Algorithm and Modified Manhattan grid topology was compared with of the Particle Swarm Optimization (PSO) and Fisheye algorithms. Table 3 shows the simulation parameters.

**Table 3:** Simulation parameter.

Sl. no	Simulation parameter	Value
1	X-limit	300, 1000, and 5000 km
2	Y- limit	20 feet road
3	Road limit	20feet road

The simulation scenario comprises 20 feet of the road with two horizontal and vertical roads. Based on the coverage distance and speed of vehicles, the number of attackers can be detected. Table 2 shows the calculation relates to the number of attackers with distance and speed. At the start of the simulation, signals flow in two horizontal directions. the signal routing is done through the normal traffic signal. The simulation results were obtained concerning the simulation parameter^27^. These simulation parameters are used for the evaluation of the proposed algorithm. Table 4 denotes the simulation results.

**Table 4:** Simulation results.

Parameter	Value
Simulator	Matlab R2014a
Channel type	Channel/ Wireless channel
Protocols	AODV, FISHEYE and Modified Fisheye
Simulation duration	100 s
Number of nodes	200
Transmission range	250 m
Movement model	Manhattan Grid Model
Model MAC layer protocol	802.11
Pause time (s)	0, 20, 40, 60, 80, 100
Maximum speed	30 m/s
Minimum speed	5 m/s
Packet rate	4 packet/s
Traffic type	CBR Data Payload
Payload Max of	512 bytes/packet
CBR connections	8, 25, 40

## Result and discussion

**Figure 12:**
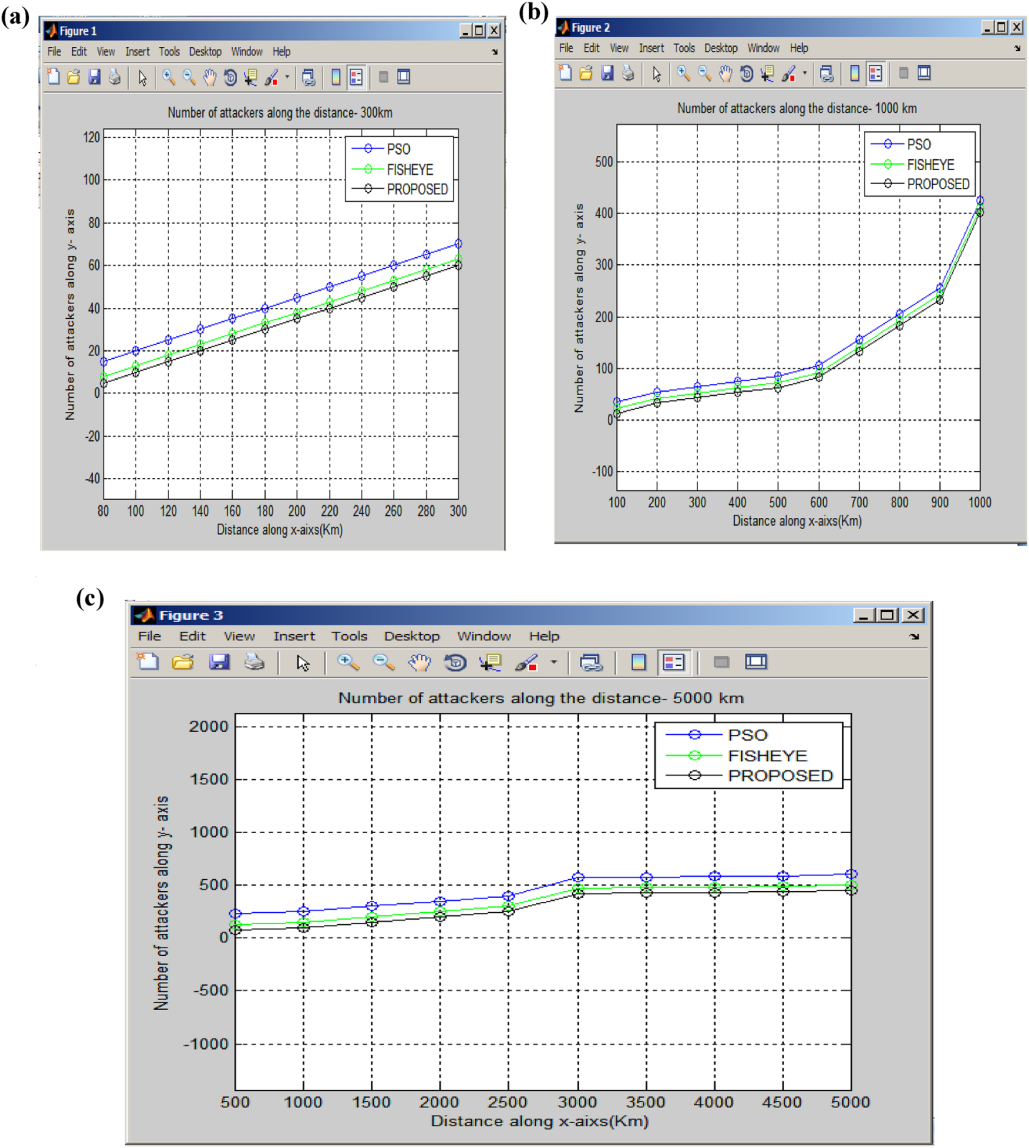
(**a**) Scenario 1: Comparison of attackers with 300 km. (**b**) Scenario: 2 Comparison of attackers with 1000 km. (**c**) Scenario 3: Comparison of attackers with 5000 km.

The modified fisheye protocol with Manhattan topology is giving better results as compared to existing algorithms in the Vehicular ad-hoc network. Figure 12a shows a particle swarm optimization (PSO) is a computational method that optimizes a problem by iteratively trying to improve a candidate (vehicle) solution concerning a given measure of quality in the communication. The movement of the coordination beam is only focused on a particular distance. The estimation of attackers in the PSO algorithm is comparatively lower than the other two algorithms for the road distance of 300 km for the modified fish eye routing algorithm. The road distance varies from 1000 km, and 5000 km long. Figure 12b,c shows that the fish eye focuses on the large distance so the Modified Fisheye routing algorithm is a proposal for an implicit hierarchical routing protocol targeted messages the node can reconstruct the whole network topology^28^.

The registration identifier and speed of the vehicle messages related to the attackers were estimated easily. The modified Manhattan grid topology reduces the number of attackers. This topology regulates the routing procedure of the vehicle, which reduces the number of attackers for a distance of 300 km, 1000 km, and 5000 km.

### Comparision scenario

The number of attackers in three scenarios was measured to provide traffic coordination in VANET and discussed in detail.

#### Comparision of attackers with 300 km

The scenario involves a 20-foot road intersected by two horizontal and vertical roads. The count of detectable attackers depends on vehicle coverage distance and speed. Existing algorithms like PSO (Particle Swarm Optimization) and FSR (Fish-eye State Routing) show higher attacker counts compared to the proposed MFSR (Modified Fish-eye Routing) algorithm. When the maximum coverage distance is 300 km, PSO detects 70 attackers, FSR detects 62, while the Modified Fish-eye Routing algorithm, implemented using a Manhattan grid topology, identifies a reduced count of 60 attackers, which is lower than both existing algorithms.

#### Comparision of attackers with 1000 km

The proposed algorithm, MFSR (Modified Fish-eye Routing), shows a lower count of attackers compared to existing algorithms such as PSO (Particle Swarm Optimization) and FSR (Fish-eye State Routing). In the implementations of PSO and FSR algorithms, the count of attackers is 425 and 405, respectively, within a maximum coverage distance of 1000 km. However, when employing the Modified Fish-eye Routing algorithm with a Manhattan grid topology, the reduced count of attackers is 399 within a coverage distance of 1000 km, which is lower than the counts observed in the existing algorithms.

#### Comparision of attackers with 5000 km

The proposed algorithm, MFSR (Modified Fish-eye Routing), records a lower count of attackers in comparison to established algorithms like PSO (Particle Swarm Optimization) and FSR (Fish-eye State Routing). Within the PSO and FSR algorithm implementations, the count of attackers reaches 630 and 500, respectively, within a maximum coverage distance of 5000 km. However, by implementing the Modified Fish-eye Routing algorithm utilizing a Manhattan grid topology, the reduced count of attackers stands at 440 within a coverage distance of 1000 km, showing a decrease compared to the counts observed in the existing algorithms.

The proposed Modified Fisheye State Routing with Manhattan Grid topology gives better results compared to the existing PSO and Fisheye Routing Algorithms. The proposed Modified Fisheye State Routing(MFSR) algorithm reduces the number of attackers to 30% compared with the existing PSO and Fisheye algorithms for road distances of 300 km,1000 km, and 5000 km. The limitation in implementing the proposed system in actual traffic environments is Security and Privacy. Vehicle communication systems gather substantial data, posing vulnerability to exploitation if adequate security measures are not enforced. Another disadvantage is the smart city framework needs to address the specific data latency and throughput requirements of individual applications.

### Case study: traffic at road intersection area

**Figure 13:**
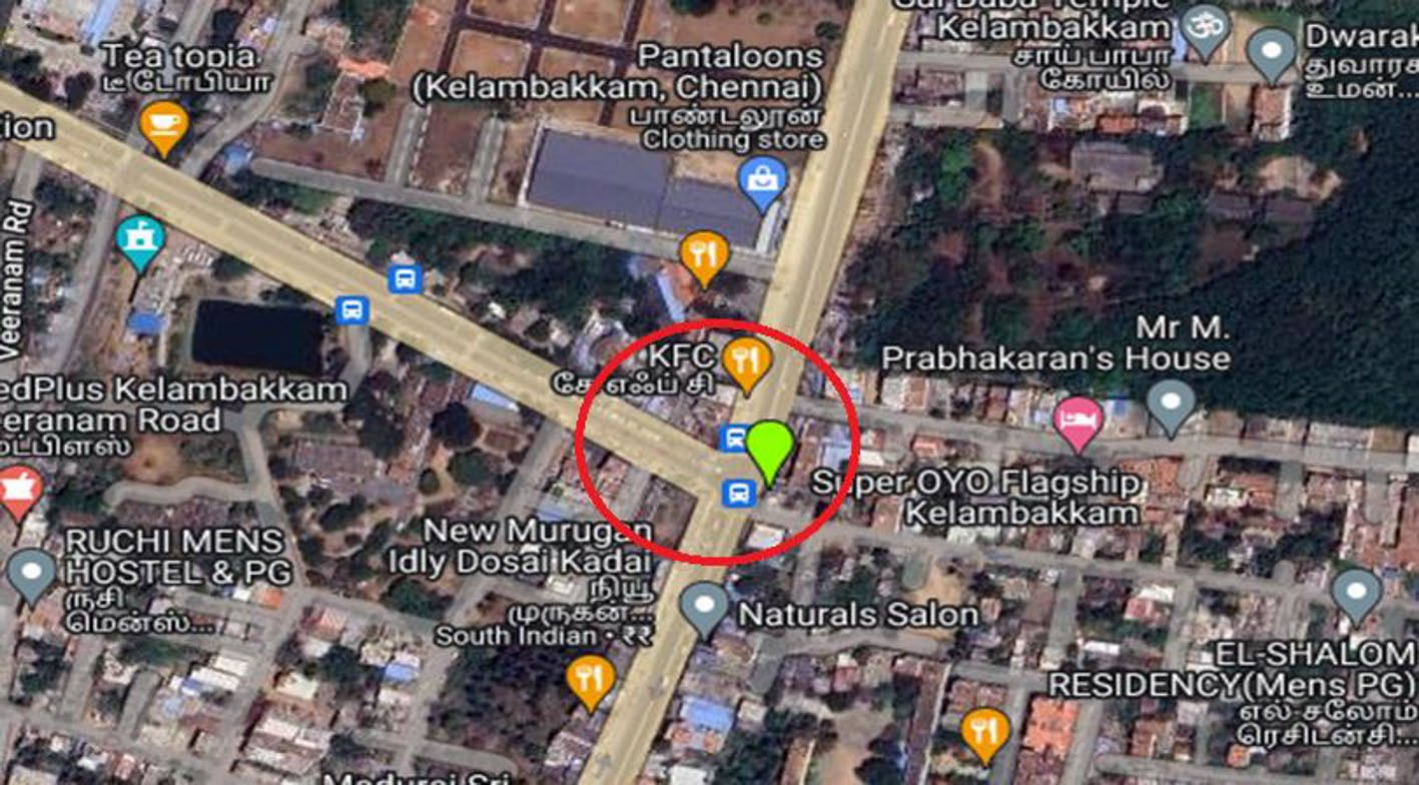
Road Intersection at Kelambakkam in Chennai [https://www.google.com/maps/d/edit?mid=13SPDzezn4_3GRBUaIECZO9SGDqo7miI&ll=12.790314921286466%2C80.22169960000001&z=17].

As urban traffic increases, congestion becomes a critical issue, particularly at intersections where multiple roads converge, allowing vehicles to navigate in different directions. For traffic engineers in urban settings, studying intersections holds significant importance. In Kelambakkam, a town located in Chennai, there exists a T-shaped intersection involving three roads: one leading towards Sholinganallur in the north, another towards Vandalur in the west, and the third directing traffic southbound to Kelambakkam and Thiruporur. Understanding and analyzing this Kelambakkam intersection is pivotal for traffic management and urban planning purposes. The location of Kelambakkam in Chennai and the study intersection at Kelambakkam is shown in Fig. 13.

**Figure 14:**
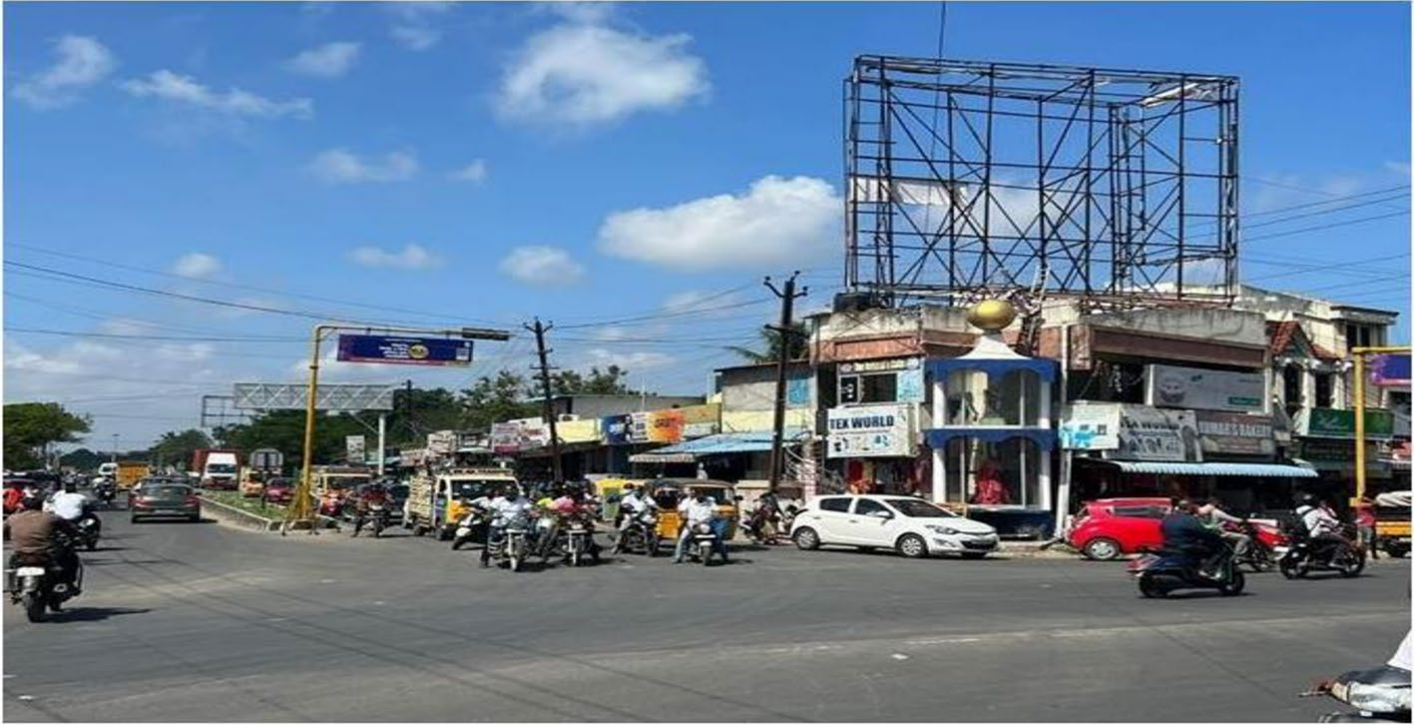
Congested flow at Kelambakkam intersection.

The region is conveniently reachable via Metropolitan Transport Corporation buses and features a moderately sized bus terminus located in close proximity to Kelambakkam market . The Congested flow at Kelambakkam intersection is shown in the Fig. 14.

The analysis encompasses a traffic volume count survey. At the Kelambakkam intersection, vehicles traveling in various directions share the same space simultaneously, further compounded by 1800 pedestrians per hour who also require that same space for crossing maneuvers.

## Conclusion

This research work primarily focuses on enhancing road safety and minimizing injuries within the context of mixed traffic conditions on undivided roads.The study's main objectives revolve around the utilization of innovative techniques, including the Modified Manhattan grid topology and the modified fisheye algorithm. The Modified Manhattan grid topology is implemented to guide drivers effectively on undivided roads, promoting safer navigation. Furthermore, the utilization of the Fuzzy C-Means algorithm serves the purpose of detecting the count of potential jamming attackers, while the modified fisheye algorithm is instrumental in minimizing the data exchanged among vehicles. Subsequently, the formulation of the Particle Swarm Optimization (PSO) algorithm is employed to derive more precise coordinates for jamming attackers within individual clusters. To assess the validity of the results, the Fuzzy C-Means algorithm is employed, revealing a noteworthy 30% decrease in the number of attackers, and achieving a commendable 70% accuracy in challenging traffic scenarios. These findings hold promise for the continued development and deployment of Intelligent Transportation Systems in similar dynamic traffic environments worldwide. In the future, We can extend traffic prediction and control by using Artificial intelligence techniques. Additionally, our aim involves utilizing a simulator to simulate realistic transportation system scenarios integrated with V2X communication within smart cities.

## Data Availability

The datasets used and/or analysed during the current study available from the corresponding author on reasonable request.
